# Targeting ATF5, CEBPB, and CEBPD with Cell-Penetrating Dpep Sensitizes Tumor Cells to NK-92MI Cell Cytotoxicity

**DOI:** 10.3390/cells14090667

**Published:** 2025-05-02

**Authors:** Qing Zhou, Markus D. Siegelin, Lloyd A. Greene

**Affiliations:** Department of Pathology and Cell Biology, Vagelos College of Physicians and Surgeons, Columbia University Irving Medical Center, New York, NY 10032, USA; qz2266@cumc.columbia.edu (Q.Z.); ms4169@cumc.columbia.edu (M.D.S.)

**Keywords:** NK cells, NK-92MI cells, cancer, cell-penetrating, peptide, ATF5, CEBPB, CEBPD, apoptosis

## Abstract

Natural killer (NK) cells are an important innate defense against malignancies, and exogenous sources of NK cells have been developed as anti-cancer agents. Nevertheless, the apparent limitations of NK cells in clearing cancers have suggested that their efficacy might be augmented by combination with other treatments. We have developed cell-penetrating peptides that target the transcription factors ATF5, CEBPB, and CEBPD and that promote apoptotic cancer cell death both in vitro and in vivo without apparent toxicity to non-transformed cells. We report here that one such peptide, Dpep, significantly sensitizes a variety of tumor cell types to the cytotoxic activity of the NK cell line, NK-92MI. Such sensitization requires pre-exposure of tumor cells to Dpep and does not appear due to effects of Dpep on NK cells themselves. Our findings suggest that Dpep acts in this context to lower the apoptotic threshold of tumor cells to NK cell toxicity. Additionally, while Dpep pre-treatment does not prevent tumor cells from causing NK cell “inactivation”, it sensitizes cancer cells to repeated rounds of exposure to fresh NK cells. These findings thus indicate that Dpep pre-treatment is an effective strategy to sensitize cancer cells to the cytotoxic actions of NK cells.

## 1. Introduction

Natural killer (NK) cells represent an important innate organismal defense against tumor cells by promoting their apoptotic death [1,2,3,4]. To harness this response for experimental as well as potential clinical application, several human NK cell lines have been generated [1,2,3,4]. Among the most widely employed of these is the NK-92 line which in turn has been genetically modified by transfection/infection to enhance its utility and efficacy [5,6]. The NK-92MI line, in particular, was generated to produce hIL-2 so as to be independent of this interleukin for growth and cytotoxic activity [7,8].

Given that NK cell lines may be expanded in culture under appropriate conditions, can be engineered to generate a variety of CAR-NK lines, and retain the capacity to kill tumor cells, they have been identified as potential “off the shelf” therapeutics for the treatment of both solid and liquid cancers [5,9,10]. Consequently, such lines are currently in clinical trials for a variety of malignancies [1,2,3,4,5,6,11]. However, despite their promise, challenges remain, and trials thus far have shown limited efficacy [4,11,12]. One approach to address this issue is to combine NK cell-based therapy with treatments that sensitize tumor cells to their actions. While there has been some progress in this realm [13,14,15,16,17], there remains the need for combination candidates that can be used to co-target a range of cancer types, that are safe, and that have substantial anti-cancer activity on their own without compromising NK cell function or survival.

We have developed a set of cell-penetrating peptides that target the transcription factors ATF5, CEBPB, and CEBPD by associating with their leucine zipper domains and that thereby block their obligate dimerization required for activity [18,19,20]. Multiple studies (reviewed in references [18,19,20]) have shown that these factors play roles in the formation, survival, growth, and metastasis of a wide variety of cancer types and that interfering with their activities by our peptides leads to apoptotic death of malignant cells [18,19,20]. Moreover, the peptides selectively kill tumor cells and appear to have a high degree of safety in vivo [18,19]. Given these properties, we assessed the combined treatment of cancer cells with NK-92MI cells and one such peptide, designated as Dpep [19]. Dpep is a linear peptide that contains an N-terminal cell-penetrating penetratin sequence followed by a portion of the leucine zipper sequence of human CEBPD. The peptide evokes apoptotic death of a range of tumor cell types both in vitro and in vivo and has no apparent cytotoxic effects on non-transformed cells or on bodily tissues [19]. The aim of our study was to determine whether the combination of Dpep and NK-92MI cells would lead to enhanced killing of tumor cells that might ultimately lead to a therapeutic advantage. We report that pre-treatment of a variety of tumor cell types with Dpep significantly sensitizes them to killing by NK-92MI cells.

## 2. Materials and Methods

### 2.1. Cell Cultures

HCT116, MDA-MB-231, T98G, A375, A549, and MCF7 cell lines were obtained from and authenticated by the ATCC (Manassas, VA, USA) and cultured as previously described [21]. NK-92MI cells were obtained from the ATCC (#CRL-2408) and cultured in MyeloCult™ H5100 medium (STEMCELL Technologies, Cambridge, MA, USA; #05150) containing hydrocortisone (STEMCELL Technologies, Cambridge, MA, USA; #74142) and 12.5% FBS.

A375 Dpep-responsive cells and A375 Dpep-resistant cells were isolated from subcutaneous A375 cell xenografts produced in SCID mice as previously described [19]. In the case of responsive cells, the tumor-bearing mice were treated 3×/week intraperitoneally with 100 µL/mouse of 10% glycerol in PBS. For Dpep-resistant cells, tumor-bearing mice were treated 3×/week with 20 mg/kg of Dpep via intraperitoneal injection for 4 weeks at which time the tumor volumes, which had previously been near stable, began to increase. Tumors were harvested at 300–400 mm^3^ for resistant lines and 700–800 mm^3^ for responsive lines, excised, and stripped of surrounding tissues. To minimize stromal cell contamination, only the dense central portions of the tumors were selected, avoiding the peripheral regions. Tumors were minced into ~1 mm^3^ fragments, washed in DMEM containing 2.5% FBS, filtered through a 0.45 µm steel mesh to create a single-cell suspension, and further dissociated by repeated aspiration with 1 mL micropipettes. Cells were plated on dishes pre-coated with Poly-D-Lysine, which were incubated for 4 h at 37 °C, washed with PBS, and allowed to dry. The plates were then coated with 2.5% FBS containing 2 µg/cm² laminin (Sigma-Aldrich, St. Louis, MO, USA; #L2020) and incubated overnight in a humidified incubator at 37 °C with 5% CO_2_. Before seeding, excess laminin was removed, and the plates were rinsed twice with PBS. Cells were cultured in DMEM supplemented with 2.5% FBS, with the medium replaced every 4 days. After about 10 days, when colonies became substantial, cells were digested with 0.25% trypsin, resuspended, and redistributed. The medium was changed to DMEM containing 10% FBS, and cultures were passaged 3–4 times to ensure consistency before experiments.

With the exception of NK-92MI cells as described above, cultures were routinely maintained in DMEM supplemented with 10% FBS and 100 U/mL penicillin–streptomycin at 37 °C in a 5% CO_2_ atmosphere.

All lines were examined using a Universal Mycoplasma Detection Kit (ATCC, Manassas, VA, USA; #30-1012K) and verified to be free from mycoplasma contamination.

### 2.2. Peptides and Reagents

Dpep and mutated peptides, provided as acetate salts, were purchased from AlanScientific (Gaithersburg, MD, USA) with the following sequences:

Dpep: RQIKIWFQNRRMKWKKLVELSAENEKLHQRVEQLTRDLAGLRQFFK;
Dpep-mut: RQIKIWFQNRRMKWKKLVEGSAENEKGHQRVEQGTRDGAGGRQFFK

Peptides were dissolved in 10% glycerol in PBS (pH 7.2) and stored as 2 mM aliquots at −80 °C until further dilution for experimental use.

### 2.3. Cell Viability and Experimental Designs

#### 2.3.1. Cell Viability Assays on Tumor Cells

HCT116, MDA-MB-231, T98G, A375, A549, MCF7, A375 Dpep-responsive, and A375 Dpep-resistant cells were seeded in 96-well plates, with 0.1 mL of DMEM supplemented with 10% FBS in each well. After overnight incubation under standard culture conditions, the medium was replaced with DMEM containing 2% FBS and the designated concentrations of Dpep or Dpep-mut. The cells were then cultured for an additional 2 days or as indicated. NK-92MI cells were added in 0.1 mL of suspension in the same medium at the specified effector-to-target (E:T) ratio (in which NK-92MI cells represent the Effector (E) cells and tumor lines represent the Target (T) cells), and additional medium and Dpep or Dpep-mut were added to maintain the specified peptide concentrations and ensure that all wells had the same total volume of medium. The effector and target cells were cocultured for the indicated periods.

Cell viability was assessed through cell counting using either a hemocytometer or a Countess II automated cell counter (Life Technologies, Carlsbad, CA, USA). Prior to counting, the non-adherent NK-92MI cells were removed from the cultures by two washes with cold PBS. Visualization of the washed cultures by high power microscopy verified NK-92MI cell removal. Counts were carried out by a single blinded observer. Three technical repeats were carried out for each sample, and 3 independent samples were counted per condition. Fragmented cells and any remaining of the much smaller NK-92MI cells were excluded from the counts.

#### 2.3.2. Effect of Dpep on NK-92MI Cell Viability and Cytotoxic Activity

To assess whether the peptide might affect NK-92MI cell viability, NK-92MI cells were seeded in MyeloCult™ H5100 medium into 96-well plates in triplicate, treated with or without 20 or 40 µM Dpep for 24 or 96 h, and subsequently assessed for cell number.

To evaluate whether Dpep directly affects NK-92MI cell cytotoxic activity, the cells were seeded in MyeloCult™ H5100 medium in 96-well plates in 0.1 mL, with or without 20 µM Dpep, and incubated for 24 h. Following pre-treatment, A375 cells were added as a 0.1 mL suspension at a 1:1 E:T ratio. Additional Dpep was included to ensure a final peptide concentration of either 0 or 20 µM in the cocultures.

#### 2.3.3. Effect of Secreted Factors of NK-92MI Cytotoxic Activity

To investigate whether Dpep-pre-treated tumor cells enhance NK-92MI killing activity through secreted factors, A375 cells were seeded into 96-well plates with or without 20 µM Dpep and cultured for 48 h. The conditioned media were collected, centrifuged at 300× *g* for 5 min to remove cell debris, and the resulting supernatant was added to newly seeded A375 cells with or without coculture with NK-92MI cells at a 1:1 E:T ratio. For fresh cells treated with non-pre-treated conditioned medium, 20 µM Dpep was added as needed to match the Dpep concentration of fresh A375 cells treated with medium conditioned in presence of Dpep. After 24 h, cell viability was assessed.

#### 2.3.4. NK-92MI Cell Inactivation Studies

To assess whether the observed loss of cytotoxicity by NK-92MI cells after prolonged culture with tumor cells reflects their “inactivation”, A375 cells were seeded into 96-well plates at 0.1 mL per well and treated with 20 µM Dpep for 48 h. NK-92MI cells were then added as a 0.1 mL suspension at a 1:1 E:T ratio for coculture with A375 cells for an additional 8 h, with the Dpep concentration maintained at 20 µM. Subsequently, NK-92MI cells were collected, washed to remove A375 cells and residual Dpep, and added to freshly seeded A375 cells at a 1:1 E:T ratio. For comparison, the same number of fresh NK-92MI cells were also added to fresh A375 cultures. After 16 h of coculture, cell counting was performed.

#### 2.3.5. Tumor Cell Resistance to NK-92MI Cell Cytotoxicity

To investigate the possible resistance of tumor cells to NK-92MI cytotoxicity, A375 cells were seeded into 96-well plates at 0.1 mL per well and treated with 20 µM Dpep for 48 h. NK-92MI cells were then added at E:T ratios of 1:1 or 2:1, followed by incubation for 16 h before cell counting. For serial treatments with NK-92MI cells, NK-92MI cells were added to Dpep-pre-treated A375 cultures at a 1:1 E:T ratio and incubated for 8 h. Subsequently, fresh NK-92MI cells were added again at a 1:1 E:T ratio, and incubation continued for another 8 h before cell counting. Additional Dpep was included to ensure a final peptide concentration of either 0 or 20 µM in the cocultures.

### 2.4. Detection of Apoptosis by Flow Cytometry

Apoptosis in control and Dpep/NK-92MI-treated cells was assessed using the BD Pharmingen FITC Annexin V Apoptosis Detection Kit I (BD Biosciences, Franklin lakes, NJ, USA, #556547), following the manufacturer’s instructions with modifications optimized for experimental conditions.

A375 cells were seeded at a density of 5 × 10^5^ cells/mL in 6-well plates and treated with or without 20 µM Dpep for 48 h. NK-92MI cells were then added at a 1:1 E:T ratio and cocultured for an additional 24 h. After incubation, the culture medium containing non-adherent NK-92MI cells was removed and discarded. A375 cells were then washed twice with cold PBS to remove debris and remaining NK-92MI cells (confirmed by high power microscopy), detached using 0.25% trypsin-EDTA, and neutralized with FBS-containing DMEM. Cells were pelleted by centrifugation at 300× *g* for 5 min at 4 °C, washed twice with cold PBS, and resuspended in 1× Binding Buffer at a concentration of 1 × 10^6^ cells/mL.

For staining, 100 µL of the cell suspension was transferred to a 5 mL culture tube, followed by the addition of 5 µL FITC Annexin V and 5 µL PI (propidium iodide). The mixture was gently vortexed and incubated for 15 min at 25 °C in the dark. After incubation, 400 µL of 1× Binding Buffer was added to each tube, and samples were analyzed by flow cytometry within 1 h at the Columbia HICCC flow Cytometry Core.

FITC Annexin V fluorescence was detected in the FL1 channel (530/30 nm bandpass filter), and PI fluorescence was detected in the FL2 channel (585/42 nm bandpass filter). A minimum of 10,000 events per sample was acquired, and compensation was applied using single-stained controls to correct for spectral overlap. Due to the marked difference in size between A375 and NK-92MI cells, gating was performed based on forward scatter (FSC) and side scatter (SSC) properties. On an FSC-H vs. SSC-H dot plot, a polygon gate was drawn around the high-FSC/high-SSC population to selectively include the larger A375 cells while excluding the significantly smaller NK-92MI cells, which exhibit low FSC and SSC values.

### 2.5. Independence Assessment

To assess whether the observed responses to combined treatments (such as Dpep and NK-92MI cells) were the result of the independent actions of each or reflected sensitization by either agent on the response to the other, we computed values for a model in which the two treatments acted independently and compared them with the observed data. The Bliss independence model [22] was used to calculate the percentage of survival anticipated if the two treatments (A and B) acted independently when combined and was computed as the product of the mean cell survival observed for treatment A alone and the mean cell survival observed for treatment B alone and was expressed as a percentage. The SEM for the calculated value was determined as the square root of (SEM A)^2^ + (SEM B)^2^. The observed and calculated values were compared using the two-tailed Student’s *t*-test (GraphPad version 10.4.2).

### 2.6. Plate-Seq Data Analysis

Data from a previously performed Plate-seq analysis [21] were examined for differentially expressed genes (Log2FC ≤ −0.5 and ≥ 0.5, FDR ≤ 0.05) whose products are reported to affect NK cell function [21]. All raw and processed data associated with the latter study are available at the Gene Expression Omnibus under accession number GSE244579.

### 2.7. Statistical Analyses

All individual experiments were independently conducted in triplicate, and the data were presented as mean ± standard error of the mean (SEM). Statistical significance was determined using a two-tailed Student’s *t*-test (GraphPad version 10.4.2). A *p*-value below 0.05 was regarded as statistically significant.

## 3. Results

### 3.1. Dpep Does Not Adversely Affect NK-92MI Cell Growth/Survival

**Figure 1:**
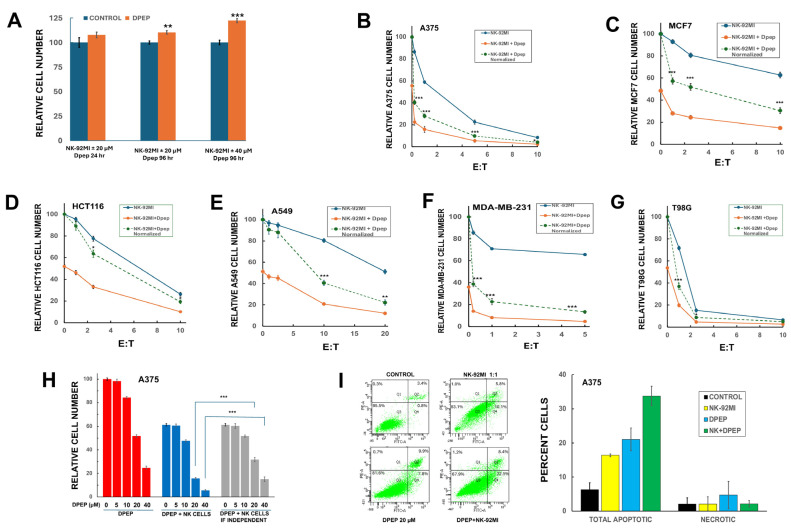
Dpep sensitizes tumor cell lines to NK-92MI cell cytotoxicity. (**A**) Dpep does not adversely affect NK-92MI cell growth/survival. Replicate NK-92MI cell cultures were treated ± 20 or 40 µM Dpep for 24 or 96 h as indicated and assessed for cell numbers. Data for treatment with 20 µM Dpep for 24 or 96 h are pooled from 3 independent experiments, each carried out in triplicate. Data for treatment with 40 µM Dpep for 24 or 96 h are pooled from 2 independent experiments, each carried out in triplicate. Values here and in subsequent panels are expressed as mean ± SEM. ** *p* < 0.005; *** *p* < 0.0005 for comparison of cultures treated ± Dpep under otherwise comparable conditions (unpaired *t*-test). (**B**–**G**) Dpep sensitizes multiple cancer cell lines to NK-92MI cell cytotoxicity. Replicate tumor cell cultures were pre-treated ± Dpep for 48 h and then exposed to the indicated E:T ratios (in which NK-92MI cells represent the Effector (E) cells and tumor lines represent the Target (T) cells) of NK-92MI cells under the same conditions for an additional 24 h as indicated. Cultures were then washed free of NK-92MI cells and assessed for numbers of surviving tumor cells. Data are plotted both to indicate relative survival under each treatment condition (solid lines) as well as in a form in which the Dpep + NK-92MI data were “normalized” (dashed lines) so that the values seen with Dpep alone (that is, without NK-92MI cells present) were set to 100. Such normalization thus provides a dose–response for the action of NK-92MI cells on Dpep-treated cells that can be compared with that for cells not exposed to Dpep. Data are pooled from 2 independent experiments, each carried out in triplicate with the exception of A549 cells exposed to NK-92MI cells as an E:T ratio of 20:1, in which the data are from 1 experiment carried out in triplicate. * *p* < 0.05; ** *p* < 0.005; *** *p* < 0.0005 for comparison of “normalized” data vs. data for NK-92MI cell treatment without Dpep at the same E:T ratio (unpaired *t*-test). (**H**) Dose–response for Dpep-promoted sensitization to NK-92MI cell cytotoxicity. A375 cells were exposed to 0–40 µM Dpep for 48 h and then for an additional 24 h with or without NK-92MI cells (E:T = 1:1) under the same conditions. Relative cell numbers are shown for each condition along with computed values (gray bars) if Dpep and NK-92MI cells acted independently without sensitization. Data were pooled from 3 independent experiments, each carried out in triplicate. *** *p* < 0.0005 for comparison of observed effects of Dpep + NK-92MI treatment vs. that calculated if the two treatments acted independently. (**I**) Assessment of apoptotic and necrotic cell death of A375 cells after no treatment (Control), 72 h treatment with 20 µM Dpep, or 72 h treatment with Dpep with the addition of NK-92MI cells (1:1) for the final 24 h, all as described in the Materials and Methods. The left-hand charts shows the flow cytometry results from one experiment. The right-hand graph shows the total percents of cells undergoing apoptosis and necrosis under each condition. The values in the graph are averages from 2 independent experiments. The error bars represent the range.

Dpep targets a wide range of tumor cell types, and because NK-92 cells were derived from a patient with non-Hodgkin’s lymphoma [5], we first assessed whether the peptide might affect NK-92MI cell growth/survival. Accordingly, replicate NK-92MI cultures were treated with or without 20 or 40 µM Dpep for up to 96 h and then evaluated for cell number. While treatment with these concentrations of Dpep reduces the survival/growth of a variety of established tumor lines by 50–90% [19], in contrast, such treatment caused no reduction in NK-92MI cell numbers, and, intriguingly, there were significant increases in cell numbers after 96 h of treatment at both concentrations of the peptide (Figure 1A).

### 3.2. Dpep Sensitizes Multiple Tumor Cell Lines to NK-92MI Cell Killing

We next asked whether Dpep exposure sensitizes tumor cell to killing by NK-92MI cells. Six diverse human tumor lines were used: HCT116 (colon cancer), MDA-MB-231 (triple negative breast cancer), T98G (glioblastoma), A375 (melanoma), A549 (lung cancer), and MCF7 (breast cancer). In each case, the cancer cells were pre-exposed ± Dpep (20 µM) for 48 h and then to various E:T (effector-to-target) ratios of NK-92MI cells for an additional 24 h in the continued presence of Dpep. Under these conditions, Dpep treatment alone decreases cancer cell number by about 50–60% (Figure 1B–G). To discern Dpep-dependent sensitization to NK-92MI cell killing, data were plotted both to indicate relative survival under each treatment condition (solid lines) as well as in a form in which the Dpep + NK-92MI data were “normalized” (dashed lines) so that the values seen with Dpep alone were set to 100 (Figure 1B–G). This normalization thus provides a dose–response for the action of NK-92MI cells on Dpep-treated cells that can be compared with that for cells not exposed to Dpep. As anticipated, each cell line showed a different and characteristic level of sensitivity to NK-92MI cells alone. Significantly, comparison of the responses of tumor cells to NK-92MI cells ± Dpep treatment shows sensitization in each case. The degree of sensitization caused by Dpep treatment varied between cell lines and as a function of the E:T ratio (Figure 1B–G). For example, there were relatively large shifts in sensitivity for A375, MDA-MB-231, MCF7, and A549 cells, and a lesser shift with T98G and HCT116 cells. However, in all cases, the combined actions of Dpep and NK-92MI cells were significantly greater than if their actions were independent.

### 3.3. Dpep Sensitizes Tumor Cells to NK-92MI Cells in a Dose-Dependent Manner

To determine the dose range at which Dpep sensitizes tumor cells to killing by NK-92MI cells, A375 cultures were pre-treated with various concentrations of Dpep for 48 h, exposed to NK-92MI cells for 24 h in the continued absence or presence of Dpep at an E:T ratio of 1:1, and then assessed for cell numbers. To highlight sensitization to NK-92MI cytotoxicity by Dpep, the observed effects were compared with those computed if the effects of the two treatments were independent (Figure 1H). The data reveal that Dpep causes relatively little sensitization to NK-92MI cells at levels that cause less than 20% cell death when applied alone, and that sensitization increases markedly as the Dpep concentration is further elevated (Figure 1H). Thus, at least for A375 cells, Dpep sensitizes tumor cells to NK-92MI cells at doses that promote >20% cell death when applied alone.

Both Dpep and NK cells promote the apoptotic death of cancer cells. To confirm that cell death with the combined treatment is also apoptotic, we treated A375 cultures as detailed above and then employed flow cytometry after Annexin V/PI staining to quantify apoptotic and necrotic cells with appropriate gating to exclude the much smaller NK-92MI population (Figure 1I). As anticipated, both treatments applied alone triggered apoptotic death. Pre-treatment with Dpep followed by the addition of NK-92MI cells also resulted in apoptotic death that was substantially enhanced compared with either agent alone. Moreover, there was relatively little change in the necrotic population under all tested conditions.

### 3.4. Sensitization of Tumor Cells to NK-92MI Cells Requires Active Peptide

**Figure 2:**
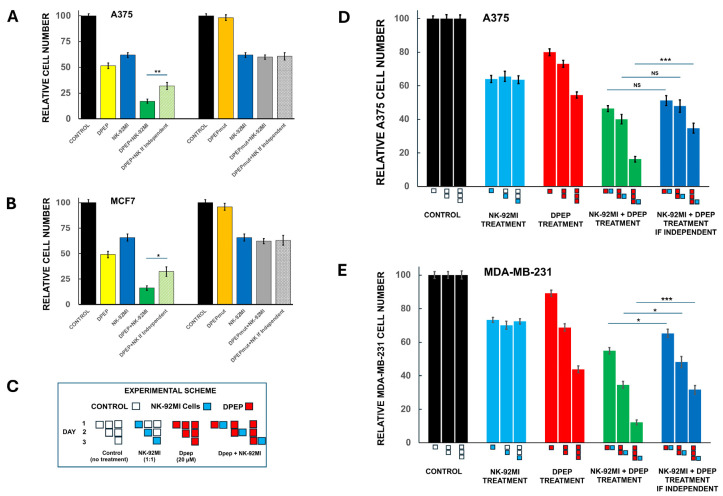
Sensitization of tumor cells by Dpep to NK-92MI cell cytotoxicity requires active peptide and increases with time of pre-treatment. (**A**,**B**) Inactive peptide does not sensitize to NK-92MI cell cytotoxicity. A375 (**A**) and MCF7 (**B**) cells were pre-treated for 2 days without (control) or with either 20 µM Dpep or a mutant inactive peptide Dpep-mut and then for an additional 24 h with NK-92MI cells (E:T 1:1 for A375 cells and 10:1 for MCF7 cells) under the same conditions. Observed cell numbers were compared with those anticipated if the actions of the two treatments were independent. Data were pooled from two independent experiments, each carried out in triplicate. Asterisks here and as follow indicate *p* values as described in the legend to Figure 1. (**C**) Scheme of experiment designed to assess the degree of sensitization to NK-92MI cell conferred by various times of pre-treatment with Dpep. (**D**,**E**) Sensitization of tumor cells to NK-92MI cells increases as a function of Dpep pre-treatment time. A375 (**D**) and MDA-MB-231 (**E**) cultures were treated as described in panel C and then assessed for cell numbers. Observed cell numbers were compared with those anticipated if the actions of the two treatments were independent. Data were pooled from two independent experiments, each carried out in triplicate. NS = not significant.

We next considered the possibility that Dpep might sensitize tumor cells to NK-92MI cell cytotoxicity via non-specific, off-target interactions or by altering cellular uptake (for example, of granzymes) due to the presence of the cell-penetrating domain. To test this, we utilized a mutant form of the peptide (Dpep-mut) in which the key lysine residues of the leucine zipper motifs were replaced with glycines. Past studies have shown that such a mutated peptide has little if any cytotoxic activity on tumor cells but should retain the capacity for cell penetration [19]. A375 and MCF7 cells were accordingly pre-treated ± Dpep or Dpep-mut for 2 days and then for an additional day with NK-92MI cells. To assess the outcome, observed cell numbers were compared with those that would be anticipated if the combined effects of NK-92MI cells and peptide were independent (Figure 2A,B). For Dpep, the peptide alone killed about half of the tumor cells in each line and for the combination with NK-92MI cells, the degree of killing was about twice that expected if the two treatments acted independently. In contrast, Dpep-mut showed very little killing and in combination with NK-92MI cells, the observed killing was very similar to that seen with NK-92MI cells alone and to the computed effect of the two together if independent. These findings thus indicate that sensitization to NK-92MI cell cytotoxicity by Dpep is not due to non-specific actions of the peptide.

### 3.5. Sensitization of Tumor Cells to NK-92MI Cells Increases as a Function of Dpep Pre-Treatment Time

In the sensitization experiments presented thus far, tumor cells were pre-treated with Dpep for 48 h and then incubated with NK-92MI cells for an additional 24 h in the presence of the peptide. This raised the question of whether the observed sensitization requires pre-treatment with the peptide for this time, or if no or less pre-treatment is required. To assess this, A375 and MDA-MB-231 cultures were pre-treated with Dpep for 0, 1, or 2 days before the introduction of NK-92MI cells for an additional day and then monitored for cell numbers (Figure 2C–E). For comparison, replicate cultures were untreated or treated with either Dpep or NK-92MI cells alone as indicated in Figure 2C. Results from the various pre-treatment conditions were compared with those anticipated if Dpep and NK-92MI cells acted independently. In the case of A375 cells, significant sensitization to NK-92MI cytotoxicity required 2 days of Dpep pre-treatment (Figure 2D). For MDA-MB-231 cells, a small but significant sensitization was seen without pre-treatment (Figure 2E). This effect was somewhat greater after 24 h of Dpep pre-treatment and markedly more pronounced after 48 h of pre-treatment. These findings thus indicate that sensitization of tumor cells by Dpep to NK-92MI cytotoxicity increases substantially over at least two days of pre-exposure to the peptide with an onset that appears to vary to some degree depending on cell context.

### 3.6. Medium Conditioned by Dpep-Treated Tumor Cells Does Not Affect NK-92MI Cell Cytotoxicity

**Figure 3:**
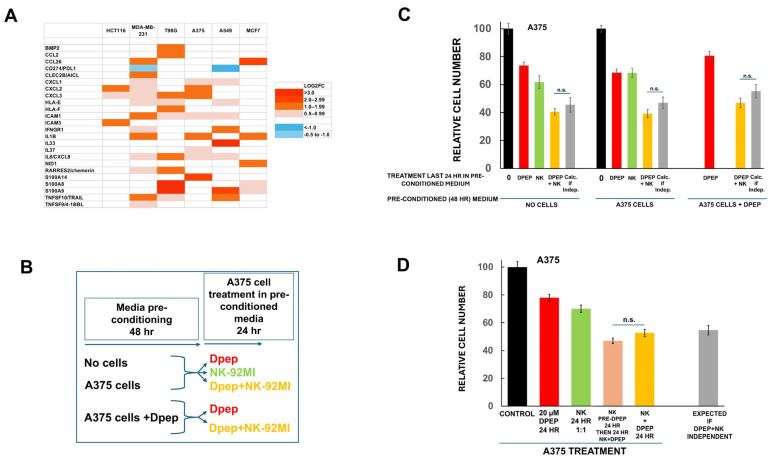
Effects of Dpep-conditioned tumor cell medium and direct treatment with Dpep on NK-92MI cell cytotoxicity. (**A**) Effect of Dpep on expression by multiple cancer cell lines of transcripts encoding proteins reported to affect NK cell function. Data are derived from a previously reported Plate-seq study [21]. (**B**) Scheme of experiment to assess the effects of media conditioned by Dpep-treated A375 cells on NK-92MI cell cytotoxicity. (**C**) Effect of media preconditioned by Dpep-treated A375 cells on NK-92MI cell cytotoxicity. Experiment was carried out as in panel (**B**). Data were pooled from two independent experiments, each carried out in triplicate. (**D**) Effect of Dpep treatment on NK-92MI cell cytotoxicity. NK-92MI cells were pre-incubated ± 20 µM Dpep for 24 h and then compared for their cytotoxic activity on A375 cells in the presence of Dpep for an additional 24 h. Data were pooled from two independent experiments, each carried out in triplicate. n.s. = not significant here and in subsequent figures.

The tumor microenvironment includes multiple secreted proteins that alter NK cell activity [23,24,25]. Examination of Plate-seq data from a past study [21] indicates that tumor cells exposed to Dpep for 48 h show cell-context-dependent regulation of a number of genes whose products are reported to affect NK cell function (Figure 3A). In the case of A375 cells, for example, these include upregulated *CXCL1*, *CXCL2*, *IL1B*, and *S100A14*. To assess whether Dpep-pre-treated cells might enhance NK-92MI cell cytotoxic activity via secreted materials (and thus appear to become sensitized to their actions), we generated media preconditioned by 48 h exposure to no cells, A375 cells, or A375 cells treated with 20 µM Dpep. Fresh A375 cultures were then subjected to 24 h of exposure to these preconditioned media with or without NK-92MI cells, and cell numbers were assessed (Figure 3B,C). The findings were compared with cell numbers anticipated if NK-92MI cells and Dpep acted independently. As anticipated, there was only a small, but statistically unsignificant, level of sensitization for cells in non-conditioned medium (Figure 3C). Similar results were observed with A375-preconditioned medium as well as with medium preconditioned by Dpep-treated A375 cells (Figure 3C). These findings thus indicate that sensitization to killing by NK-92MI cells is not due to substances released from Dpep-treated tumor cells.

### 3.7. Direct Treatment of NK-92MI Cells with Dpep Does Not Substantially Affect Their Activity

In the experiments presented thus far, NK-92MI cell exposure to Dpep was in the context of coculture with tumor cells for 24 h. This raised the possibility that Dpep might directly enhance the cytotoxic actions of NK-92MI cells under these conditions. To test this, NK-92MI cells were pre-incubated with or without 20 µM Dpep for 24 h and then compared for their cytotoxic activity on A375 cells cultured in presence of Dpep for 24 h (Figure 3D). There was only a small, but not statistically significant (*p* = 0.08) effect of Dpep pre-treatment on NK-92MI cell cytotoxicity. Thus, sensitization to NK-92MI cell killing appears to be largely independent of the direct actions of Dpep on NK cells.

### 3.8. Tumor Cell Sensitization to NK-92MI Cells Requires Susceptibility to Dpep Killing

**Figure 4:**
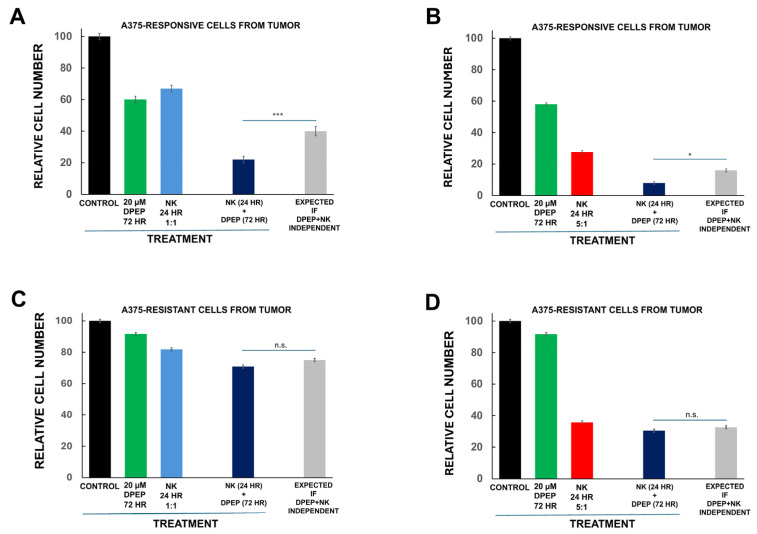
Tumor cell sensitization to NK-92MI cells by Dpep requires susceptibility to Dpep killing. (**A**) Cells isolated from a mouse A375 tumor and that are responsive to Dpep were cultured as indicated and then assessed for cell numbers. Observed results are compared with those expected if the actions of NK-92MI cells and Dpep were independent and indicate sensitization to NK-92MI cell cytotoxicity by Dpep. Data were pooled from two independent experiments, each carried out in triplicate. (**B**) As in panel (**A**), except that the E:T ratio was 5:1. (**C**) As in panel (**A**) except that the cells were isolated from a mouse A375 tumor that became resistant to Dpep-promoted cell death. (**D**) As in panel (**C**) except that the E:T ratio was 5:1. * *p* < 0.05; *** *p* < 0.0005.

One potential interpretation of our findings is that Dpep sensitizes tumor cells to NK-92MI cells by enhancing their vulnerability to apoptotic death. To test this idea, we used a multiclonal line of A375 cells (A375_Dpep-resistant1_) that was generated from a mouse xenograft tumor that became resistant to Dpep after repeated treatments with the peptide and that shows little Dpep-promoted cell death in culture. As a control, we also generated a multiclonal line (A375_Dpep-responsive1_) from a mouse A375 xenograft tumor that was never exposed to Dpep treatment and that is sensitive to Dpep-promoted cytotoxicity in culture. Both lines were pre-treated with 20 µM Dpep for 2 days and then exposed to NK-92MI cells (at E:T ratios of 1:1 and 5:1) for an additional day in the continued presence of Dpep (Figure 4A–D). The responsive A375_Dpep-responsive1_ line showed levels of cell death similar to that of parental A375 cells when treated with Dpep or NK-92MI cells alone (Figure 4A,B). It also exhibited a degree of Dpep-promoted sensitization to NK-92MI cells comparable to that found for parental A375 cells. The A375_Dpep-resistant1_ line, in contrast, showed marked resistance to Dpep-promoted killing (Figure 4C,D). It was also less responsive to NK-92MI cytotoxicity, although there was a considerable response at an E:T ratio of 5:1. Significantly, at both E:T ratios, Dpep did not sensitize the A375_Dpep-resistant1_ cells to NK-92MI cytotoxicity. These findings indicate that resistance to the apoptotic actions of Dpep correlates with the absence of Dpep-promoted sensitization to NK-92MI cell killing, thus supporting the idea that sensitization is dependent on Dpep-promoted activation of an apoptotic program.

### 3.9. Dpep and NK Cell Inactivation

**Figure 5:**
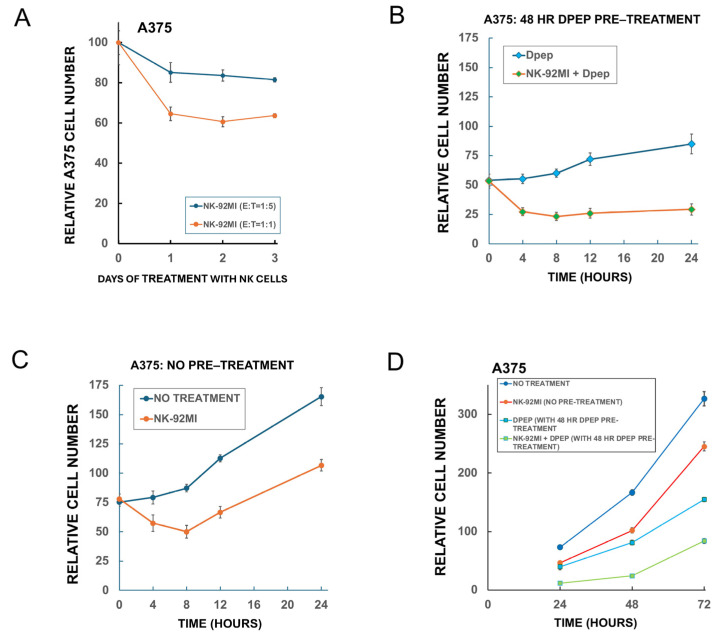
Time course of NK-92MI cell cytotoxicity on A375 cells and of sensitization by Dpep. (**A**) Time course of NK-92MI effect on A375 cell numbers at E:T ratios of 1:1 and 1:5. At each time point, the numbers of cells in treated cultures were normalized to the cell numbers in untreated cultures (set to 100). Data are from an experiment carried out in triplicate. (**B**) Time course of A375 cell growth with either no treatment or exposure to NK-92MI cells (initial E:T ratio = 1:1). Relative total cell numbers are shown. Data are from an experiment carried out in triplicate. Similar results were achieved in an independent repeat experiment. (**C**) Time course of growth of A375 cells after 48 h of pre-treatment with 20 µM Dpep and then cultured for the indicated times with either 20 µM Dpep or 20 µM Dpep + NK-92MI cells (initial E:T ratio = 1:1). Relative total cell numbers are shown. Starting cell numbers were the same as for the experiment shown in panel (**B**). Data are from an experiment carried out in triplicate. Similar results were achieved in an independent repeat experiment. (**D**) Extended time course for growth of A375 cells in the continued presence of NK-92MI cells ± Dpep pre-exposure. A375 cells were pre-treated for 48 h ± 20 µM Dpep and then exposed or not, as indicated, to NK-92MI (initial E:T ratio = 1:1) under the same culture conditions. Cell counts on replicate cultures were then carried out 24, 48, and 72 h following NK-92MI cell addition. Data are from an experiment carried out in triplicate. Similar results were achieved in an independent repeat experiment. We next probed whether NK-92MI killing activity might recover after a more prolonged exposure to tumor cells or to Dpep. Accordingly, replicate A375 cultures with or without 48 h of Dpep pre-treatment were cultured with or without NK-92MI cells and maintained under the same conditions for 3 days and monitored for cell number over time (**D**). Although NK-92MI-containing cultures started at reduced numbers of A375 cells due to the initial killing actions of the NK-92MI cells, comparison of the growth rates up to 72 h indicated that there was no resumption of their cytotoxic activity.

One feature of NK cells is that prolonged exposure to tumor cells can cause them to lose killing efficacy [26,27,28]. Indeed, when A375 cells were exposed to NK-92MI cells for 1, 2, and 3 days at E:T ratios of 1:5 and 1:1, the killing activity largely took place only during the first day (Figure 5A). To more closely examine the time course of NK-92MI cell cytotoxicity on tumor cells under the conditions of our experiments and the effects of Dpep pre-treatment and presence, A375 cells (±48 h of Dpep pre-treatment) were exposed to NK-92MI cells for 4, 8, 12, or 24 h and then assessed for cell numbers (Figure 5B,C). The findings indicate that maximal cytotoxicity with NK-92MI cells occurred by about 4–8 h of exposure, irrespective of Dpep pre-treatment/presence. Without Dpep pre-treatment, after 8 h of NK-92MI cell exposure and the consequent reduction in cell numbers, the A375 cells commenced to increase in number at a rate similar to that in untreated cultures (Figure 5B). For A375 cells pre-treated and maintained with Dpep, the increase in cell number over the course of the experiment (compared with non-treated cells) was substantially attenuated as anticipated due to cell death and suppression of proliferation [19] (Figure 5C). For the condition in which Dpep-pre-treated cells were exposed to NK-92MI cells + Dpep, there was very little recovery of cell numbers over 24 h, thus substantiating the enhanced efficacy of the combined treatments.

**Figure 6:**
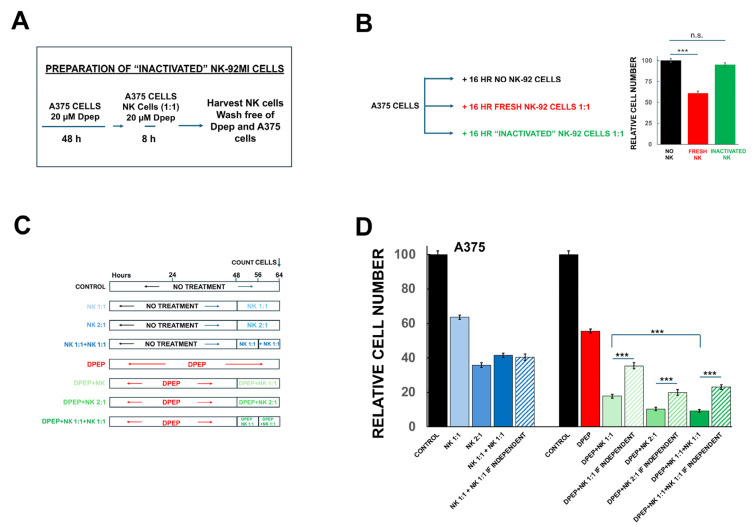
Dpep does not affect NK-92MI cell inactivation but sensitizes tumor cells to serial treatment with fresh NK-92MI cells. (**A**) Scheme for preparation of inactivated NK-92MI cells by exposure to Dpep pre-treated tumor cells in presence of Dpep. (**B**) NK-92MI cells become inactivated by exposure to Dpep-pre-treated tumor cells in presence of Dpep. The scheme of the experiment is indicated at the left with results shown on the bar graph. Data are pooled from 2 independent experiments, each carried out in triplicate. (**C**) The scheme of the experiment to assess whether Dpep pre-treated tumor cells respond to serial treatments with NK-92MI cells. (**D**) Dpep-pre-treated A375 cells respond to serial treatments with NK-92MI cells. A375 cells were treated as shown in panel (**C**) and as indicated on the graph. Data are pooled from 2 independent experiments, each carried out in triplicate. *** *p* < 0.0005.

The above findings indicate that NK-92MI cells eventually lose cytotoxic activity after exposure to tumor cells in both the presence and absence of Dpep. However, it was unclear whether the sensitization of tumor cells to NK-92MI cytotoxicity might be due in part to effects of Dpep on the degree and rate of appearance of NK-92MI cell inactivation. To address this, we next generated NK-92MI cells that were cocultured with Dpep-treated A375 cells for 8 h, the time at which cytotoxic activity appears to be lost after tumor cell exposure in absence of Dpep, and then washed them free of the attached A375 cells and of Dpep (Figure 6A). These preconditioned NK-92MI cells as well as fresh NK-92MI cells were then incubated with fresh A375 cells for an additional 16 h and subjected to cell counts (Figure 6B). In contrast to non-conditioned NK-92MI cells, the A375/Dpep-preconditioned NK-92MI cells showed little cytotoxic activity against the tumor cells and thus appeared to have undergone inactivation similar to that of NK-92MI cells without Dpep exposure.

### 3.10. Dpep-Treated Tumor Cells Respond to Serial Treatments with NK-92MI Cells

The observation that NK-92MI-promoted cytotoxicity appears to occur for only a limited period of time, both with or without sensitization by Dpep, raised the question of whether this effect reflects not only NK-92MI cell inactivation but also resistance of the tumor cells to NK-92MI cell killing. To assess this, A375 cells with or without Dpep pre-treatment were subjected to a second exposure to fresh NK-92MI cells (E:T = 1:1) after a time at which killing by the initial treatment had largely ceased (Figure 6C). We also compared the results to cytotoxicity observed when NK-92MI cells were applied at a single E:T of 2:1 (Figure 6C). For NK-92MI cells treatment alone, killing was similar for a single treatment at an E:T ratio of 2:1 and two treatments at an E:T of 1:1 (Figure 6D, blue bars). Moreover, in the serial treatment condition, the observed level of cytotoxicity was not significantly different than expected if the two treatments were independent (Figure 6D, dark blue bar vs. stripped blue bar). For tumor cells pre-treated with Dpep, as found above, single exposure to NK-92MI cells at E:T ratios of 1:1 and 2:1 showed a significant level of sensitization compared to that expected if the responses to each treatment were independent (Figure 6D, solid green vs. stripped bars). Importantly, treatment with a second, fresh dose of NK-92MI cells at an E:T of 1:1 after the first dose of cells had become exhausted produced a new, significant round of cytotoxicity to reach a level of tumor cell survival about half that observed with the first treatment at an E:T = 1:1 and similar to that achieved with a single NK-92MI cell treatment at an E:T of 2:1 (Figure 6D, green bars). Moreover, the two serial treatments showed a highly significant level of sensitization compared to that expected if the responses to Dpep and NK-92MI cells were independent. Taken together, these observations thus indicate that tumor cell resistance is not a major contributor to the loss of sensitivity to NK-92MI cells that occurs within 6–8 h. Notably, these findings further indicate that Dpep-treated tumor cells have the capacity to respond to serial treatments with NK-92MI cells and do so with unabated sensitization.

## 4. Discussion

The major aim of this work was to determine whether Dpep, a cell-penetrating peptide that targets the leucine zipper transcription factors ATF5, CEBPB, and CEBPD, could effectively sensitize tumor cells to the cytotoxic actions of NK-92MI cells. Dpep and related peptides have shown a remarkable selectivity, promoting cell death in a wide range of cancer cell types while sparing non-transformed cells both in vitro and in vivo. The NK-92 line, from which NK-92MI cells were derived, was generated from mononuclear blood cells of a patient with an NK cell lymphoma [5]. This raised the possibility that the peptide might negatively affect NK-92MI cell viability. However, Dpep did not diminish survival/growth of NK-92MI cells for up to at least 96 h of exposure and appears, rather, to somewhat enhance their numbers. Given that most of the NK-92MI cytotoxicity in the presence of Dpep occurs within the first 8 h, any potential long-term negative influences on the cells by Dpep does not appear to be a major concern. It is of further interest that although the NK-92 cell line was derived from a lymphoma patient, they have not been found to be tumorigenic when transplanted into immunocompromised mice [29]. This property is consistent with the observed absence of toxicity of Dpep on NK-92MI cells.

The findings here show that Dpep sensitizes a variety of tumor cell types to NK-92MI cell killing. Though all lines tested showed sensitization, the degree was variable. In general, those lines that are most resistant to NK-92MI cells, including MCF7, A549, and MDA-MB-231, showed the highest level of sensitization by Dpep. In each line, the degree of sensitization varied as a function of the E:T ratio and Dpep concentration. For A375 cells, sensitization was seen at Dpep concentrations that caused >20% cell death when applied alone.

One question regarding the observed sensitization by Dpep is whether this is due primarily to actions on NK-92MI cells or to actions on tumor cells. While we cannot fully rule out enhanced efficacy of NK-92MI cells under our experimental conditions, we did not find evidence for this mechanism. Pre-treatment of NK-92MI cells with Dpep did not enhance their killing activity. Dpep also did not rescue NK-92MI cells from inactivation caused by exposure to tumor cells. Additionally, although transcriptome profiling indicated that Dpep-treated tumor cells may produce secreted proteins with the capacity to alter NK cell function, medium conditioned by Dpep-treated tumor cells did not enhance NK-92MI killing activity.

In our experiments, enhanced NK-92MI cell killing increased as a function of time of tumor cell pre-treatment with Dpep and was maximal after 48 h of peptide pre-exposure. We also found that tumor cells resistant to Dpep-promoted cell death did not show Dpep-dependent sensitization to NK-92MI cells. These observations are consistent with the idea that Dpep directly sensitizes tumor cells to NK-92MI cell cytotoxicity rather than directly altering NK-92MI cell cytotoxic activity. Prior studies showed that Dpep causes apoptotic death of tumor cells [19]. The peptide increases levels of the pro-apoptotic protein BMF and substantially reduces levels of the pro-survival protein survivin in all tumor cells tested thus far [18,19,30]. In addition, Dpep causes cell-context-dependent regulation of additional genes encoding pro-apoptotic proteins [21]. One scenario is that in addition to causing outright apoptotic death of a portion of target tumor cells, Dpep pushes others towards death and that cytotoxic agents such as granzymes and death ligands released from NK-92MI cells provide the final irreversible death stimulus. Thus, by engaging apoptotic signaling, Dpep pre-treatment may reduce resistance of cancer cells to NK cell killing. Nevertheless, we cannot fully rule out the possibility that Dpep affects the expression of tumor proteins that enhance NK-92MI cell engagement and/or release of cytotoxic molecules.

NK-92 cells and lines derived from them by genetic engineering have been widely considered for clinical use [4,5,6,7,8,9,10,11], offering distinct advantages over T cell-based approaches. Unlike T cells, NK cells can nonspecifically kill tumor cells without prior sensitization and are not restricted by major histocompatibility complex (MHC) expression, which is often downregulated in cancer cells to evade T cell recognition [31]. Nevertheless, challenges remain, and trials thus far have shown limited efficacy [12]. Thus, there is a need to identify additional means to sensitize tumor cells to NK-92 cell cytotoxicity. What advantages might Dpep confer in this context? For one, unlike a number of therapeutic agents that have been tested for enhancement of NK-92 cell killing, Dpep does not appear to have major side effects, nor does it appear to be toxic to NK cells. Also, unlike CAR-NK cells which are generally produced against cancers with specific markers, Dpep affects a wide range of tumor types, and thus, its combination with NK-92MI cells represents a truly “off-the-shelf” treatment. In addition, it seems likely that Dpep will also sensitize cancers to various CAR- and otherwise modified NK-92 cells. Finally, one limiting factor of NK-92 cell efficacy in vivo is penetration into tumors [28,32]. Dpep is designed to penetrate tissue barriers and enter cells, including tumor cells. Histological examination of tumors in animals treated with Dpep indicate severe disruption of tumor integrity which would very likely enhance accessibility to NK cells [19]. Taken together, these potential advantages support the combination of Dpep with NK cell-based therapies.

In our studies, we observed a time-dependent loss of NK-92MI-promoted killing of tumor cells, both with and without Dpep pre-treatment. Such a phenomenon is similar to that previously described to occur in the tumor microenvironment, both for endogenous NK and NK-92 cells [27,33]. This did not appear to be due to tumor cell resistance to NK-92MI cells but rather to tumor cell-triggered diminution of NK cell activity. In this context, exposure to fresh NK-92MI cells elicited a new round of cytotoxicity, and this too showed sensitization by Dpep. These observations thus indicate that a therapy consisting of combined/tandem Dpep-NK-92MI cell treatment would retain efficacy over multiple exposures.

While we have used NK-92MI cells is the present work, it seems highly likely that Dpep will similarly sensitize tumor cells to other lines with the features of NK or other immune cells that promote apoptotic death of targets. Our findings also raise the possibility that Dpep will sensitize malignant cells to endogenous cytotoxic immune cells.

## 5. Limitations

There are several limitations to this study. For one, the data presented are all derived from in vitro experiments; it remains to be seen whether Dpep sensitizes tumor cells to NK-92MI cells in living animals and the most effective means of combining Dpep and NK-92MI cell treatments in vivo. Furthermore, we have not delved into the molecular mechanisms of the events described here. For instance, do NK-92MI cells show enhanced release of lytic granule contents and/or of death ligands? Additionally, this study is limited to NK-92MI cells. It would be important to determine whether Dpep sensitizes cancer cells to other NK cell lines, to endogenous NK cells, to T-cells, and/or to CAR-modified cytotoxic lymphocytes.

## 6. Conclusions and Future Directions

Our findings show that pre-treatment with Dpep, a cell-penetrating peptide designed to target the transcription factors ATF5, CEBPB, and CEBPD, sensitizes a variety of cultured cancer cells to the cytotoxic activity of NK-92MI cells. These observations raise the potential use of a combined Dpep/NK-92MI treatment in a therapeutic setting. In this context, there remain outstanding questions for future studies. For example, does such sensitization occur in vivo, and if so, would this also be the case for endogenous NK cells and other cytotoxic lymphocytes as well as for CAR-NK/CAR-T cells? Additionally, what are the molecular mechanisms that underlie the observed sensitization, and might they be harnessed to further enhance the efficacy of a combined treatment? Addressing these issues will be key to advancing the potential clinical translation of this strategy.

## Data Availability

The raw data supporting the conclusions of this article will be made available by the authors on request.

## Abbreviations

The following abbreviations are used in this manuscript:

NK cells	Natural killer cells
CAR-NK	Chimeric Antigen Receptor-engineered NK cells
FBS	Fetal bovine serum
E:T	Effector-to-target
FITC	Fluorescein isothiocyanate
PI	Propidium iodide
